# Complete Genome Sequences of Mycobacterium tuberculosis Isolates Subjected to 200 Continuous Passages

**DOI:** 10.1128/MRA.00356-20

**Published:** 2020-06-25

**Authors:** Lucio Vera-Cabrera, Carmen A. Molina-Torres, Jose Marco Lopez-Ortiz, Jorge Castro-Garza, Carlos Cordova-Fletes, Jorge Ocampo-Candiani

**Affiliations:** a Laboratorio Interdisciplinario de Investigación Dermatológica, Servicio de Dermatología, Hospital Universitario “Dr. José Eleuterio González,” Universidad Autonoma de Nuevo Leon, Monterrey, NL, México; b Centro de Investigación Biomédica del Noreste, Instituto Mexicano del Seguro Social, Monterrey, NL, México; c Departamento de Bioquimica, Facultad de Medicina, Universidad Autonoma de Nuevo Leon, Monterrey, NL, México; Broad Institute

## Abstract

We report 6 draft genome sequences corresponding to Mycobacterium tuberculosis H37Rv and M. tuberculosis DR689, a Beijing isolate, plus their counterparts subjected to 200 continuous passages in Middlebrook 7H9 broth, either alone or with ox bile.

## ANNOUNCEMENT

Tuberculosis is still a public health problem worldwide (1). In Mexico, the number of new pulmonary and meningeal tuberculosis cases has increased in the last several years (2). New drugs and immunoprophylactic media are needed to control this disease. Considering that continuous passaging produces important biological changes in microorganisms (3, 4), we decided to subculture Mycobacterium tuberculosis isolates. Although M. tuberculosis and M. bovis belong to the same bacterial complex, they have important biological differences (5).

In our project, we subcultured isolates of different lineages of M. tuberculosis (6), including MTBH37Rv and a Beijing isolate, DR689. The only isolate that has already been sequenced is strain H37Rv, which constitutes the reference genome sequence used worldwide (GenBank accession number NC_000962.3). This strain was donated to J.C.-G. in 1998 by Fred Quinn, when he was a research microbiologist at the Tuberculosis and Mycobacteriology Branch, Division of AIDS, STD, and TB Laboratory Research, Centers for Disease Control and Prevention (Atlanta, GA). It was cryopreserved at −70°C until we started the subculture. Both strains MTBH37Rv and MTBDR689 were subjected to 200 continuous passages on M7H9-Tween 80-oleic acid-albumin-dextrose-catalase (OADC); because Calmette and Guerin used ox bile to better suspend the bacteria, we processed some samples with and some without bile. We use the following nomenclature to refer to our cultures: *M. tuberculosis* H37Rv parental strain (MTBH37RvP), without bile (MTBH37Rv-P200) and with bile (MTBH37Rv-P200B), and *M. tuberculosis* DR689 parental strain (MTBDR689P) without bile (MTBDR689-P200) and with bile (MTBDR689P-200B).

DNA was extracted from the colonies grown on Lowenstein-Jensen agar using the cetyltrimethylammonium bromide (CTAB)-NaCl technique with modifications described previously (6). Subsequently, high-throughput sequencing was carried out using the Illumina MiSeq genome analyzer (San Diego, CA, USA). Sequencing libraries were prepared using the NuGEN Ultralow v2 kit or the Nextera XT v2 kit following the manufacturer’s recommended protocol. The libraries were sequenced on a NextSeq 500 instrument using midoutput v2 chemistry (2 × 150 bp) or on a MiSeq instrument using V2 chemistry (2 × 250 bp); the fastq reads obtained were mapped against the M. tuberculosis H37Rv reference genome sequence reported in GenBank (accession number NC_000962.3) using the BWA-MEM aligner (7) from the Sequencher v5.4.6 suite (Gene Codes, Ann Arbor, MI). Default parameters were used except where otherwise noted.

The genomes were assembled into contigs using SPAdes Genome Assembler v3.9.0 and deposited in GenBank. Annotations were made using the single Prokaryotic Genome Annotation Pipeline (PGAP) of the NCBI (8).

### Data availability.

This whole-genome shotgun project has been deposited at DDBJ/EMBL/GenBank under the following accession numbers: VRNC00000000 (Mycobacterium tuberculosis H37RvP), VRND00000000 (Mycobacterium tuberculosis H37Rv-P200), VRNE00000000 (Mycobacterium tuberculosis H37Rv-P200B), VRNF00000000 (Mycobacterium tuberculosis DR689P), VRNG00000000 (Mycobacterium tuberculosis DR689-P200), and VRNH00000000 (Mycobacterium tuberculosis DR689-P200B). All these sequences belong to BioProject accession number PRJNA559369. The characteristics of the draft genomes are summarized in Table 1.

**TABLE 1:** Draft genome characteristics and accession numbers of M. tuberculosis H37RvP, M. tuberculosis DR689P, and their counterparts, subjected to 200 continuous passages with or without bile

Strain/isolate	GenBank accession no.	SRA accession no.	Genome size (Mbp)	G+C content (%)	No. of contigs	*N*_50_ value (bp)	No. of reads	Coverage (×)	No. of CDSs^*a*^	% coding proteins	No. of tRNAs	No. of rRNAs
*M. tuberculosis* H37RvP	VRNC00000000	SRX8374041	4.3	65.55	103	142,434	1,690,242	162	5,489	98.2	42	1
*M. tuberculosis* H37Rv-P200	VRND00000000	SRX8374042	4.3	65.55	155	64,842	7,851,072	162	4,378	97.1	45	1
*M. tuberculosis* H37Rv-P200B	VRNE00000000	SRX8374043	4.3	65.43	205	57,433	3,899,892	109.5	4,622	93.5	46	1
*M. tuberculosis* DR689P	VRNF00000000	SRX8374038	4.3	65.59	115	105,204	1,373,090	34	4,464	95.8	49	1
*M. tuberculosis* DR689-P200	VRNG00000000	SRX8374039	4.3	65.44	230	45,562	2,040,850	58	4,523	94.2	45	1
*M. tuberculosis* DR689-P200B	VRNH00000000	SRX8374040	4.3	65.56	160	64,269	5,448,990	81	4,359	96.4	47	1

a CDSs, coding DNA sequences.
